# Effects of Uric Acid on Diabetes Mellitus and Its Chronic Complications

**DOI:** 10.1155/2019/9691345

**Published:** 2019-10-13

**Authors:** Qing Xiong, Jie Liu, Yancheng Xu

**Affiliations:** ^1^Department of Endocrinology, Zhongnan Hospital of Wuhan University, Wuhan, Hubei 430071, China; ^2^Department of Endocrinology, Affiliated Haikou Hospital of Xiangya Medical College, Central South University, Haikou, Hainan 570208, China

## Abstract

With the deepening of the researches on uric acid, especially in the study of metabolic diseases, uric acid has been found to be closely related to obesity, metabolic syndrome, nonalcoholic fatty liver disease, diabetes, and other metabolic diseases. Uric acid causes a series of pathophysiological changes through inflammation, oxidative stress, vascular endothelial injury, and so on and thus subsequently promotes the occurrence and development of diseases. This review confirmed the positive correlation between uric acid and diabetes mellitus and its chronic complications through the pathogenesis and clinical studies aspects.

## 1. Introduction

In recent years, human intake of foods such as those with the umami flavor (rich in purines), high added sugar (sucrose), and high fructose corn syrup have increased dramatically [1]. Fructose is the main component of added sugar. Unlike other sugars, fructose can cause mitochondrial oxidative stress [2, 3] and inhibits AMPK [4], and the subsequent intracellular ATP depletion [5] and nucleotide turnover lead to a significant increase in serum uric acid [6]. In addition to causing gout, many studies have shown that hyperuricemia is also closely related to cardiovascular diseases, metabolic syndrome, insulin resistance, and diabetes [7, 8]. However, its function is a matter of debate [9]. Here, we reviewed the effects of hyperuricemia on diabetes and its complications and concluded that high levels of uric acid is closely related to diabetes and its chronic complications.

### 1.1. Uric Acid Formation

**Figure 1:**
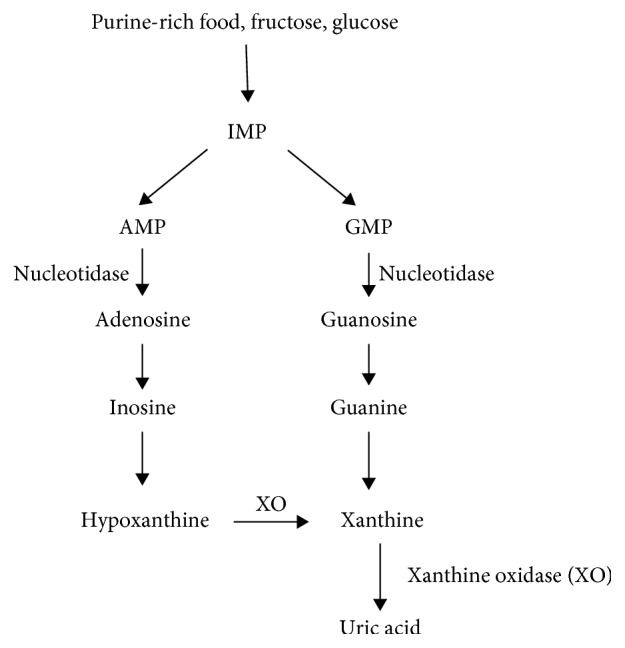
Process of purine metabolism in humans.

In the human body, uric acid is the ultimate product of purine metabolism (Figure 1 [10]). It is generated in the liver. Purine nucleotides decompose to hypoxanthine and guanine, some of which can be recycled and phosphorylated into hypoxanthine nucleotides, while the remaining part is metabolized by xanthine dehydrogenase/oxidase (XDH/XO) enzymatic reaction to the terminal product uric acid. XDH/XO is mainly expressed in the parenchymal cells of the liver and small intestine. XDH has low reactivity and can be converted to XO. Uric acid production primarily depends on the amount of substrate and the activity of XO [11]. In the end, XDH/XO promotes the final steps in purine metabolism which convert hypoxanthine to xanthine and xanthine to UA [11]. The kidney also plays an important role in the regulation of blood uric acid levels. The circulating uric acid is easily filtered from the glomeruli into the renal tubule. About 90% of filtered UA is reabsorbed by the middle of the proximal convoluted tubule mainly by urate transporter 1 (URAT1) and glucose transporter 9 (GLUT9) [12], and the remaining excreted 10% is responsible for 60–70% of total body uric acid excretion [13, 14]. A small amount of uric acid secreted in the intestine is responsible for 30–40% [14]. The production and excretion rate of uric acid is relatively constant in healthy people. Changes in the uric acid content in body fluids can reflect the state of metabolism, immunity, and other functions of the human body. If the body produces too much uric acid or the excretion mechanism is degraded, the body will retain excessive uric acid. Hyperuricemia was defined as the circulating uric acid levels of more than 5.7 mg/dl for women and 7.0 mg/dl for men [15]. When the blood uric acid concentration exceeds the norm, the human body fluid becomes acidic, which affects the normal function of the human cells, subsequently leading to metabolic disease in the long term [16–18].

## 2. Pathological Mechanism of Uric Acid on Diabetes and Its Chronic Complications

### 2.1. Uric Acid and Diabetes

**Figure 2:**
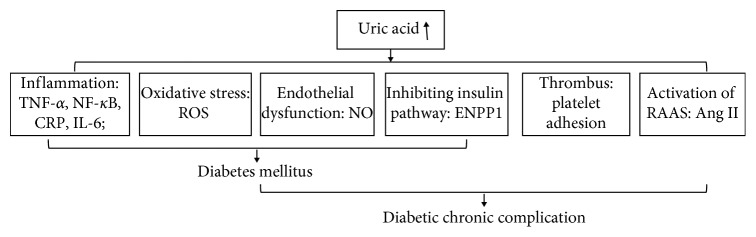
Metabolism of uric acid leading to diabetes mellitus and its chronic complication.

At present, many studies have shown that the relevant pathological mechanisms include some aspects as follows (Figure 2):

1. *Inflammation*. Increased uric acid levels in the blood promoted the expression of interleukin-1*β* (IL-1*β*), interleukin-6 (IL-6), tumor necrosis factor-*α* (TNF-*α*) [19], and CRP production [20]. In animal studies, the activation of inflammation induced by UA decreases insulin sensitivity in mice [21], and infusion of UA into mice can increase TNF-*α* levels and activate the classical inflammatory pathway [22]. In human studies, serum UA was positively associated with TNF-*α*, interleukin-6 and C-reactive protein in healthy people [23].
2. *Oxidative Stress*. Excessive uric acid will lead to an increase in reactive oxygen species (ROS) production, which leads to inflammation and dysfunction in the vessel [24]. UA is a powerful antioxidant that can remove superoxide and hydroxyl radicals in plasma, and UA has prooxidant effects in vascular tissue by increasing ROS production, such as H_2_O_2_ [24]. UA-mediated oxidative stress-induced lipid peroxidation, DNA damage, and activation of inflammatory factors finally lead to cellular damage [24]. Oxidative stress also can affect the expression of insulin gene, causing a decrease in insulin secretion [25].
3. *Endothelial Dysfunction*. Endothelial dysfunction is characterized by deficiencies in the synthesis and/or bioavailability of endothelium-derived NO [26]. In addition, UA reduces endothelial NO bioavailability in humans [27]. Uric acid inhibits proliferation and migration of endothelial cells and NO secretion [20]. UA can react with NO to form 6-aminouracil, UA-dependent ROS reacts with NO to form peroxynitrite, and UA can hold back L-arginine uptake and stimulate L-arginine degradation [6]. As a result of the effects of hyperglycemia and neurohormonal activation, UA levels are independently associated with endothelial dysfunction in animals and humans, thereby promoting hypertension [28].
4. *Inhibiting Insulin Pathway*. UA directly inhibits the trigger of insulin signaling pathway by an ectonucleotide pyrophosphatase/phosphodiesterase 1 (ENPP1) recruitment at the receptor level [29].

All factors interference with glucose homeostasis and insulin sensitivity promotes the development of diabetes [30–32].

### 2.2. Uric Acid and Diabetic Chronic Complications

The aforementioned changes to diabetes are also directly related to the metabolic disorder: desulfation of glycosaminoglycans (GAGs) and formation of advanced glycation end products (AGE) and receptors (RAGE) [33]. It is widely believed that polyol bypass, protein kinase C, hexosamine activation, advanced glycosylation products (AGEs), increased hyperglycemia-induced mitochondria production of reactive oxygen species (ROS), inflammation, and endothelial dysfunction are the common pathogenic characteristics of chronic complications of diabetes mellitus [10,33–39], which mainly include macroangiopathy, microangiopathy, and neuropathy. Two other mechanisms are associated with chronic complications as follows (Figure 2):

1. *Activation of RAAS*. Uric acid can lead to the activation of the renin-angiotensin-aldosterone System (RAAS), through increasing the production of juxtaglomerular renin [40]. UA-induced ROS stimulated the increase of plasma angiotensin II which induced aldosterone release, leading to activation of RAAS [24, 41]. RAAS activation induced afferent renal arteriolopathy and tubulointerstitial fibrosis in rodent models [42]. In diabetes, RAAS activation causes a range of pathological changes including vascular dysfunction, high intraglomerular pressure, inflammation, and so on, leading to cardiovascular and renal complications [43].
2. *Thrombus*. Uric acid seems to trigger platelet adhesion and aggregation, thus favoring vascular thrombosis [44].

## 3. Epidemiology Studies

### 3.1. Uric Acid and Diabetes

The relationship between uric acid and diabetes has gradually become a hot topic of research, but controversy still exists. On the one hand, some study reported uric acid was not associated with diabetes. For example, Sluijs et al. [45] used a genetic score of 24 uric acid-related sites for Mendelian randomization studies, in the European prospective survey data—Cancer and Nutrition (EPIC) study, which was an interactive case-cohort study of vast number of subjects from eight European countries. In EPIC, after a mean of 10 years of follow-up, the results suggested that hyperuricemia was not salient associated with a higher risk of diabetes after adjusting for interference factors when their participant number was increased from 10,576 to 41,508. Similarly, a large prospective cohort study was performed by Li [46] who followed up 4412 nondiabetic patients for 4.7 years to study urate changes in glucose metabolism. They found the uric acid concentration was not related to an increased risk of type 2 diabetes mellitus (T2DM).

On the opposite hand, more clinical trials demonstrated uric acid was significantly associated with diabetes. For example, Bombelli et al. [47, 48] randomly selected 3,200 northern Italian residents between the ages of 25 and 74 and found that increased uric acid resulted in an increased risk of impaired fasting glucose (IFG), and people with higher median UA levels may also develop metabolic syndrome and diabetes. In women, serum uric acid (SUA) levels in the normal range were associated with an increased risk of new-onset diabetes compared with women with low-normal values [49]. Older adults with high levels of uric acid (6.0 mg/dl for men and 5.5 mg/dl for women) were more susceptible to metabolic syndrome and T2DM, especially in the 75–84 years age group [50]. Serum UA was an important predictor of risk of metabolic syndrome, diabetes, and hypertension in adult males [51]. However, the relationship between blood UA and decreased insulin sensitivity in patients with type 1 diabetes mellitus is weaker than in healthy subjects [52].

Through reading a large number of literature and studies, we believe that uric acid is closely related to diabetes. Poor lipid metabolism in individuals with higher UA levels may lead to increased fasting and postprandial insulin levels, high-sensitivity C-reactive protein, hepatic insulin resistance index, and decreased glomerular filtration rate and skeletal muscle insulin sensitivity; high levels of SUA may impair liver insulin sensitivity and insulin clearance [53]. Perticone F [54] was documented when hypertensive NGT ≥ 155 mmHg, and UA is closely related to 1-h postload glucose during an oral glucose tolerance. We [55] analyzed the clinical characteristics and islet function index of 403 newly diagnosed patients with T2DM (mean age, 50.21 ± 13.34 years old; 62.5% male) and analyzed the SUA levels according to gender. Multivariate linear regression analysis showed that SUA had an independent effect on insulin secretion in female patients; the islet *β*-cell function of male was also affected by SUA, age, body mass index (BMI), and blood lipids; SUA correlated positively with insulin secretion and the insulin resistance index in male patients.

In terms of gestational diabetes, Leng [56] found that the SUA level is positively related with the risk of T2DM and prediabetes in the Tianjin region of China gestational diabetes mellitus (GDM) prevention planning data. In the group with GDM and impaired glucose tolerance (IGT), the mean SUA level was significantly increased in early pregnancy, and a UA level of 3.95 mg/dl could predict GDM with 60% specificity and 100% sensitivity [57].

### 3.2. Uric Acid and Diabetic Chronic Complications

#### 3.2.1. Uric Acid and Diabetic Macrovascular Disease

Diabetic macroangiopathy refers to atherosclerosis of blood vessels such as the aorta, coronary artery, basilar artery, renal artery, and peripheral arteries, especially in the heart and cerebrovascular diseases, which is caused by dysfunction of endothelial cells, advanced glycation end product (AGEs/RAGEs) system, the hexosamine pathway, inflammation, oxidant stress, protein kinase (PKC), and polyol [34–37]. Some clinical studies have shown a positive correlation between uric acid and diabetic macroangiopathy. Yan et al. [58] used Mendelian randomized analysis to determine whether there is a causal relationship between UA and diabetic macrovascular disease and found that the prevalence of diabetic macrovascular disease was significantly higher in the hyperuricemia group than in the healthy population, suggesting that UA and diabetic macrovascular disease are related. Indeed, the link between female-weighted genetic risk score (GRS) and diabetic macrovascular disease was greater than expected. Hyperuricemia was also observed to be associated with an increased incidence of atrial fibrillation in hospitalized patients with T2DM [59]. Hyperuricemia can increase the risk of sudden atrial fibrillation by approximately four-fold [60] and is associated with cardiovascular mortality [61]. Cardiovascular and cerebrovascular diseases are mainly caused by ischemia and hypoxia resulting from coronary atherosclerosis. Du et al. [62] performed a meta-analysis of patients with T2DM to determine whether SUA levels were associated with cerebral infarction and calculated the ratio of means (RoM) for SUA and the average cerebral infarction or average diabetes control ratio of individual studies and then compared it with the calculated 95% confidence intervals. The results showed that higher SUA levels might lead to cerebral infarction in patients with T2DM. Wang et al. [63] used the “Comprehensive Diabetes Prevention and Control Study (CRPCD)” data to explore the relationship between SUA and ischemic stroke in patients with T2DM in China. A total of 19,442 participants were enrolled in a cross-sectional study. The SUA level was significantly higher in patients over 60 years of age than in people under 60 years of age. Serum UA levels were independently and positively correlated with ischemic stroke in patients under 60 years of age, and it was characterized by U-type association in patients over 60 years of age. We speculated that the incidence of other established stroke risk factors such as hypertension, dyslipidemia, and chronic kidney disease increased with age would made it difficult to establish UA as an independent role in stroke.

Diabetic hyperglycemia causes metabolic abnormalities, which can affect systemic organs. Diabetic foot is caused by peripheral vascular disease, peripheral (motor, sensory, and autonomic) neuropathy, and excessive mechanical stress (repetitive external or minor trauma) in diabetic patients, leading to the destruction and deformity of the soft tissue and bone joint system of the foot [64]. The pathogenesis is partly the same as diabetic vascular and neuropathy complications [65]. Uric acid can be used as an independent risk factor to assess the development of diabetic foot [66].

#### 3.2.2. Uric Acid and Diabetic Microangiopathy

Diabetic microangiopathy is a specific complication of diabetes. The typical changes comprise microcirculatory disorders and microvascular basement membrane thickening, which mainly lead to diabetic nephropathy (DN) [51] and diabetic retinopathy (DR) [67–70].

*(1) Uric Acid and Diabetic Nephropathy*. Diabetic nephropathy is a long-standing microvascular complication of diabetes and is the leading cause of end-stage renal disease in developed countries [10, 71]. As an inflammatory factor, UA increases oxidative stress and promotes the activation of the renin-angiotensin-aldosterone system (RAAS) [21, 41]. Therefore, UA levels are associated with the occurrence and development of DN and are independent risk factors for early kidney disease [72, 73], which help to predict microalbuminuria progression [74]. Serum UA and microalbuminuria levels were significantly positively correlated with renal disease in patients with T2DM [75]. Patients with higher SUA levels have poorer renal function, independent of glycated hemoglobin (HbA1c) or the duration of diabetes [76]. In T2DM, there is an independent and significant positive association between higher blood UA and an increased risk of a reduced glomerular filtration rate (eGFR) [77]. Blood UA levels greater than 5.5 mg/dl can predict chronic kidney disease of stage 3 and above in T2DM [78]. The level of SUA that protects against progression of type 2 diabetic nephropathy (diabetic kidney disease (DKD)) is lower than the current normal value. The optimal cut-off value is 377.5 µmol/l (6.3 mg/dl) for men and 309.0 *μ*mol/l (5.2 mg/dl) for women [79]. In Chinese patients with T2DM, UA-related alleles such as *SLC2A9* rs11722228 (solute carrier family 2 member 9), *SLC2A9* rs3775948, and *ABCG2* rs2231142 (ATP binding cassette subfamily G member 2) may affect susceptibility to DKD [80]. Contrast-enhanced ultrasound (CEUS) was used to show renal microvascular hyperperfusion, with a decreased glomerular filtration rate and reduced UA excretion in patients with DKD [81]. Xanthine oxidase (XO) is a very important enzyme that is responsible for the conversion of sulfhydryl groups to UA. Elevation of UA by 1 *μ*mol/l enhanced the probability of albuminuria by 1.5%, and a rise in XO activity of 1 U/l also increased the probability of albuminuria by 1.5%. In diabetes, both XO and uric acid are independently associated with albuminuria [82].

In patients with type 1 diabetes without complications, higher UA levels are associated with lower GFR, which is due to UA-mediated increased resistance in afferent renal arteriole promoting the renal microcirculation ischemia [83, 84].

In type 1 diabetes, kidney damage is more common in men whose SUA and creatinine concentrations and the albumin excretion rate are higher than those in female patients. Indeed, hyperglycemia adversely affects the activity of estrogen receptors (ER) and this may be gender-specific. The progression of renal disease in men with T1D is associated with a decline in free estradiol levels [85], and 17*β*-estradiol shows antioxidant, antiapoptotic, and anti-inflammatory properties [86]. The SUA level in boys but not girls with T1D was positively correlated with subclinical inflammation marker levels (CRP, IL-6, TNF-*α*), renal function indicators (albumin excretion rate, cystatin-C level), and blood pressure; it was negatively correlated with anti-inflammatory IL-10 [87].

*(2) Uric Acid and Diabetic Retinopathy*. Diabetic retinopathy (DR) is a specific fundus lesion that is the main cause of blindness in patients with diabetes [88]. Based on the changes of haemodynamics or vascular geometry, vascular injury is considered to be the prime motivator for the initiation and progression of DR, including pericytosis, platelet aggregation, thickening of basement membrane, and neuroglial damage [89]. The blood retinal barrier, as precondition to vision acuity, is vulnerable to injury during the progression of DR. This is a consequence of the interplay of AGE, hexosamine, polyol, inflammation, NO decline, oxidative stress, PKC, and RAS [38]. Uric acid is closely related to these pathological changes. Clinically, DR is classified into nonproliferative diabetic retinopathy (NPDR) (also known as simple type or background type) and proliferative diabetic retinopathy (PDR), according to whether or not retinal neovascularization occurs [90]. In Chinese patients with T2DM, reduction in urinary uric acid excretion (UUAE) is an independent risk factor for DR [91]. Elevated SUA levels are significantly associated with albuminuria and DR severity [92], but not with the retinal nerve fibre layer or macular thickness [93]. A study reported that increased SUA levels were associated with an increased severity of DR in Taiwan [94]. Kuwata [95] analyzed data from 1839 patients with T2DM in Japan by gender stratification and found that higher SUA levels were associated with an increased risk of DR in men, but not in women. The results showed sex hormones play an important role in the metabolism of uric acid, which deserved to discuss the specific mechanism further.

#### 3.2.3. Uric Acid and Diabetic Peripheral Neuropathy

Diabetic neuropathy is one of the most common chronic complication of diabetes [96], characterized by damage to nerve glial cells, axons, and endothelial cells, and the morbidity from 30% to 50% in T2DM [97]. Diabetic peripheral neuropathy (DPN) is the main clinical manifestation of sensory and autonomic nerve symptoms, distal symmetry polyneuropathy, and motor neuropathy are the most common types of DPN [98]. The pathophysiology changes conclude polyol pathway, PKC activity, increased AGEs, oxidative stress (ROS), inflammation (IL-1*β*, IL-6, TNF*α*, and COX-2), microvascular alterations (endothelial dysfunction), nerve degeneration and regrowth (MMPs, Schwann cells and ECM), and the changes of the blood-nerve barrier [39, 99, 100]. Lin et al. [101] observed significant differences in the ratio of motor and sensory nerve amplitude and conduction velocity (CV) parameters between groups with different blood UA levels (both *P* < 0.05). Blood UA levels were negatively correlated with the ratio of motor and sensory nerve amplitude and CV. Blood UA at 9 mg/dl and total cholesterol of 5.2 mmol/l were significantly associated with DPN in patients who had suffered from T2DM for more than 10 years. Yu et al. [102] performed a meta-analysis of 1388 patients with T2DM with peripheral neuropathy and in 4746 patients without peripheral neuropathy and showed that SUA levels were significantly elevated in patients with diabetes complicated with peripheral neuropathy and that increased hyperuricemia was related with increased risk of peripheral neuropathy.

## 4. Conclusion

Complex genetic and environmental factors contribute to causing diabetes, and chronic complications of diabetes may occur throughout the body. The pathogenesis of T2DM is complex, involving various interacting factors. Its increased incidence rate is a great concern worldwide. Hyperuricemia is closely related to the development of diabetes and its chronic complications. Many animal and human experiments have confirmed that UA mainly affects diabetes and its complications through inflammation, oxidative stress, endothelial function damage, and other effects. We call for further researches to explore the molecular mechanism, especially in the direct effect of uric acid on insulin secretion.
